# Mid-infrared free-electron laser tuned to the amide I band for converting insoluble amyloid-like protein fibrils into the soluble monomeric form

**DOI:** 10.1007/s10103-014-1577-5

**Published:** 2014-04-24

**Authors:** Takayasu Kawasaki, Jun Fujioka, Takayuki Imai, Kanjiro Torigoe, Koichi Tsukiyama

**Affiliations:** 1 IR Free Electron Laser Research Center, Research Institute for Science and Technology (RIST), Tokyo University of Science, 2641, Yamazaki, Noda, Chiba 278-8510 Japan; 2 Department of Pure and Applied Chemistry, Faculty of Science and Technology, Tokyo University of Science, 2641, Yamazaki, Noda, Chiba 278-8510 Japan

**Keywords:** Free-electron laser, Amyloid, Aggregate, Fibrils, Refolding, Amide I band

## Abstract

A mid-infrared free-electron laser (FEL) is operated as a pulsed and linearly polarized laser with tunable wavelengths within infrared region. Although the FEL can ablate soft tissues with minimum collateral damage in surgery, the potential of FEL for dissecting protein aggregates is not fully understood. Protein aggregates such as amyloid fibrils are in some cases involved in serious diseases. In our previous study, we showed that amyloid-like lysozyme fibrils could be disaggregated into the native form with FEL irradiation specifically tuned to the amide I band (1,620 cm^−1^). Here, we show further evidence for the FEL-mediated disaggregation of amyloid-like fibrils using insulin fibrils. Insulin fibrils were prepared in acidic solution and irradiated by the FEL, which was tuned to either 1,620 or 2,000 cm^−1^ prior to the experiment. The Fourier transform infrared spectroscopy (FT-IR) spectrum after irradiation with the FEL at 1,620 cm^−1^ indicated that the broad peak (1,630–1,660 cm^−1^) became almost a single peak (1,652 cm^−1^), and the β-sheet content was reduced to 25 from 40 % in the fibrils, while that following the irradiation at 2,000 cm^−1^ remained at 38 %. The Congo Red assay as well as transmission electron microscopy observation confirmed that the number of fibrils was reduced by FEL irradiation at the amide I band. Size-exclusion chromatography analysis indicated that the disaggregated form of fibrils was the monomeric form. These results confirm that FEL irradiation at the amide I band can dissect amyloid-like protein fibrils into the monomeric form in vitro.

## Introduction

A mid-infrared free-electron laser (mid-IR FEL) is operated as a pulsed and linearly polarized laser with tunable wavelength, and it can excite specific bonds within the mid-IR region, accounting for its use in ablation of biological tissues as well as thermodynamic analyses of biomolecules [1–5]. In particular, the surgical therapy of pathological tissues can be facilitated by the FEL, since a feature of the FEL is that it causes less thermal collateral damage than other continuous-mode CO_2_ and yttrium aluminum garnet lasers [6, 7]. Investigators in Vanderbilt University have performed the laser-induced ablation of corneal tissue using Mark-III FEL, observed secondary structural changes and peptide fragmentation of collagen, and investigated the ablation spot size for the mechanism [8–10]. Further, at Duke University, the system was applied to the ablation of rat brain and the examination of laser lesions and histological assessment using laser pulses tuned to the –OH, –CH, and amide I and II bands [11]. Recently, alternative laser systems have been developed for the ablation of biological tissues because the FEL is costly and complex [6, 12]. In any case, one conclusion from these studies is that FEL irradiation can induce major changes in the higher-order structure of protein matrices. We are attempting to apply the IR free-electron laser at Tokyo University of Science (FEL-TUS) to biomedical techniques and to supply the FEL beam to biomedical users all over the world; as an application example, the amyloid aggregate was targeted [13, 14]. Amyloid proteins reported thus far can be roughly divided into two categories (Tables 1 and 2): those that are related to neurodegenerative diseases (Table 1) and those that are not (Table 2). These tables also list the frequencies of the amide I bands of those amyloid proteins. The former group includes Aβ [15, 16], tau protein [17], polyglutamine [18], transthyretin [19], prion protein [20], S100 protein [21], and α-synuclein [22]. The latter group (Table 2) contains lysozyme [23], calcitonin [24], myoglobin [25], insulin [26], and β_2_-microglobulin [27]. Interestingly, the wave numbers of the amide I of such protein aggregates are around 1,610–1,640 cm^−1^, while those of globular proteins containing α-helix-rich structures are around 1,650 cm^−1^ [28]. These red shifts are considered to be caused by the formation of an anti-parallel β-sheet structure during amyloid fibrillation, although the detailed mechanisms of formation and dissociation have not yet been disclosed. Although a relationship between the toxicity and the structural hierarchy has not been known, the amyloid structure is probably intrinsic in all proteins. Previously, we tested the FEL for dissecting the amyloid structure using lysozyme as it was commercially available and found that the β-sheet content decreased during irradiation tuned to the amide I band (1,620 cm^−1^) [14]. This result indicates that the amyloid-like lysozyme fibril is a flexible structure and can be refolded into the native state under appropriate conditions. In contrast, insulin is smaller in size than lysozyme and is barely soluble in neutral pH solution, quite different from the characteristics of lysozyme. In the present study, we tested if the FEL tuned to the amide I band could dissect the insulin fibrils into the native form similar to lysozyme fibrils.

**Table 1:** Amyloid proteins related with neurodegenerative diseases

	Major diseases	Amide I (cm^−1^)	Ref.
Aβ1-40/Aβ1-42	Alzheimer’s disease	1,630/1,635	[15, 16]
Tau	Frontotemporal dementia	1,630	[17]
Poly-glutamine	Huntington’s disease	1,625	[18]
Transthyretin	Familial amyloidotic polyneuropathy	1,615	[19]
Prion protein	Creutzfeldt-Jakob disease	1,618, 1,630	[20]
S100A6	Amyotrophic lateral sclerosis (ALS)	1,625	[21]
α-Synuclein	Parkinson’s disease	1,630	[22]

**Table 2:** Amyloid proteins not related with neurodegenerative diseases

	Amide I (cm^−1^)	Ref.
Lysozyme	1,614	[23]
Calcitonin (internal peptide)	1,639	[24]
Myoglobin	1,617	[25]
Insulin	1,628	[26]
β_2_-microglobulin (related with kidney dialysis-associated amyloidosis) (21–31 fragments)	1,629	[27]

## Materials and methods

### Materials

Phosphotungstic acid and Congo Red were purchased from Sigma-Aldrich (Tokyo, Japan). Acetic acid, human insulin, and sodium chloride (NaCl) were purchased from Wako Pure Chemical Industries (Osaka, Japan). The KBr mini-plate was purchased from Jasco Engineering Co. (Tokyo, Japan).

### Mid-infrared free-electron laser facility at the Tokyo University of Science (FEL-TUS)

**Fig. 1:**
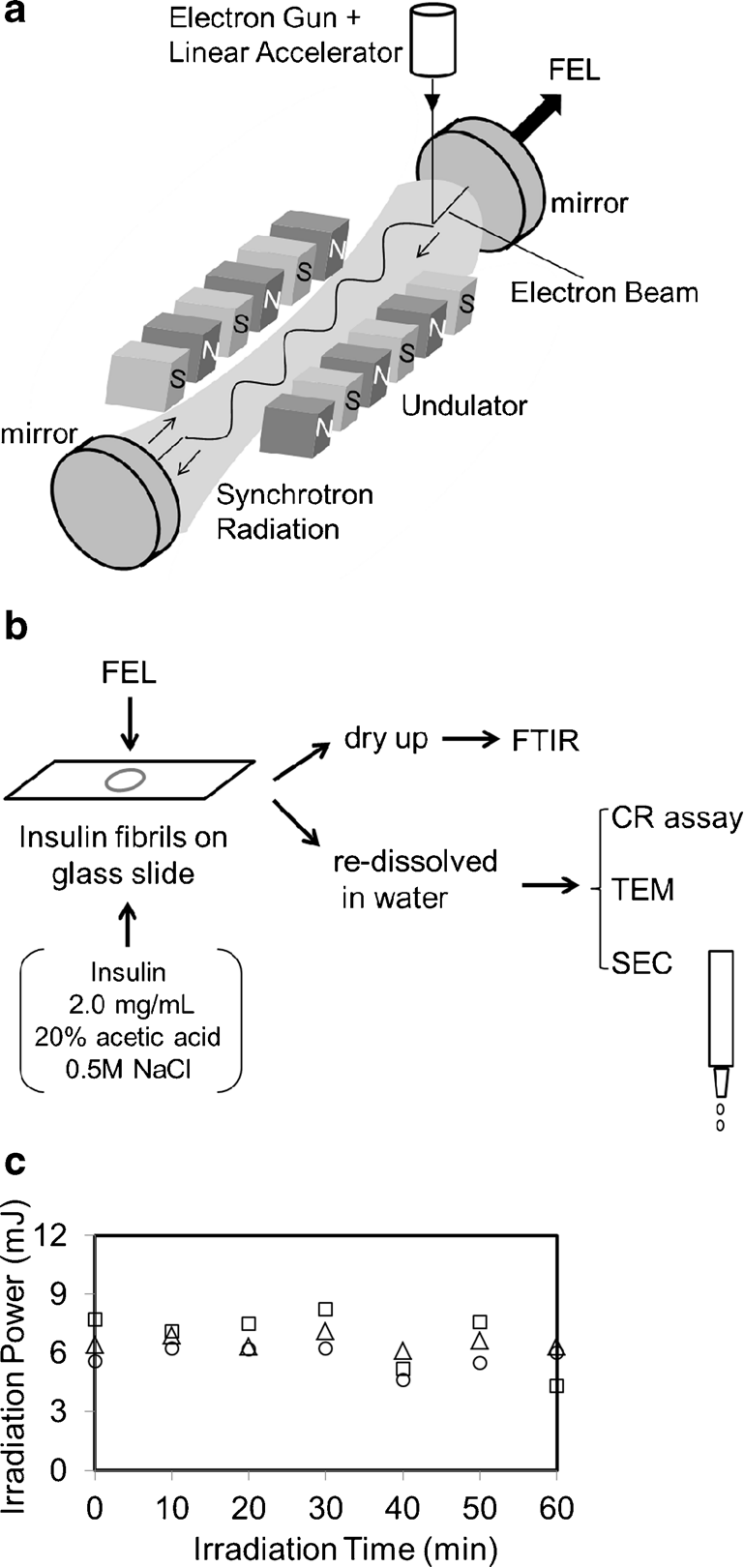
FEL irradiation method. **a** The FEL beam can be generated using an electronic gun. The electron beam is accelerated to 1.5 MeV through the linear accelerator. The periodic length of the undulator is tunable and can give various wavelengths of FELs for users. **b** The FEL beam output is transported through the vacuum tube and directed by means of the gold-coated mirror onto the sample. The insulin sample was placed within the circle of the beam spot on the glass slide and irradiated with the FEL. The sample was air dried or redissolved in water and subsequently analyzed by various methods. **c** Power monitoring during irradiation. The FEL beam was tuned to the amide I band and was directed onto the sample as described earlier. The beam power was measured at 10-min intervals for 60 min. The *triangles*, *squares*, and *circles* represent the first, second, and third experiments, respectively

The FEL-TUS can generate a laser beam using synchrotron radiation as a seed, with a variable wavelength within the mid-infrared region of 5–16 μm (625–2,000 cm^−1^) (Fig. 1a). An electron beam generated by a high-frequency RF electron gun (2,856 MHz) is accelerated by a linear accelerator and injected to an undulator (a periodic magnetic field). The electron beam is forced to oscillate in the undulator to generate synchrotron radiation (SR). Light of a specific wavelength satisfying the following equation is amplified by an interaction between the generated SR and the electron beam:

$$ \lambda =\frac{\lambda u}{2{\gamma}^2}\left(1+\frac{{\mathbf{\mathcal{K}}}^2}{2}\right) $$

In the equation, *λ* is the FEL wavelength to be amplified, *λu* is the periodic length of the undulator, *γ* is proportional to the acceleration energy of the electron beam, and *K* is proportional to the strength of the periodic magnetic field. The amplified SR is reflected upstream of the electron beam by a mirror equipped downstream of it and is re-reflected at the upstream mirror to interact with the electron beam again, which produces a coherent laser light. FEL-TUS provides two types of laser pulses, macro-pulse and micro-pulse. The macro-pulse has duration of ~2 μs and a repetition rate of 5 Hz throughout the operation, consisting of a train of micro-pulses with durations of 2 ps each. The interval between two consecutive micro-pulses is 350 ps. The energy of the laser pulse used for the current experiment was in the range of 6–8 mJ macro-pulse^−1^, which could be measured using an energy meter (SOLO2, Gentec-EO Inc., Quebec, Canada).

### Preparation of insulin fibrils and irradiation of IR FEL

Insulin powder was dissolved to a concentration of 2.0 mg/mL in H_2_O (1 mL) containing 20 % acetic acid and 0.5 M NaCl and incubated for 20 h at 37 °C. The resulting aggregates were precipitated by centrifugation at 14,000 rpm for 15 min at room temperature, washed by the addition of 0.5 mL of distilled water, and then air dried. The insulin fibrils were resuspended in water containing 20 % acetic acid on a glass slide and were irradiated with the output of the FEL tuned to various wavelengths at 37 °C. To avoid the vaporization of water, 10 μL of 20 % acetic acid was periodically added freshly to the suspension during irradiation. After the irradiation was completed, the sample on the glass was dried and subjected to various analyses (Fig. 1b).

### Fourier transform infrared spectroscopy (FT-IR)

FT-IR spectra were recorded on an FT/IR 615 spectrophotometer (Jasco International Co., Ltd., Tokyo, Japan) using a solid KBr mini-plate. The protein sample was mixed with the KBr pellet and a thin plate was prepared, and the measurements were performed using 16 scans at 4-cm^−1^ resolution. The secondary structures of the insulin samples were estimated using the bundled protein analysis software (IR-SSE; Jasco Co., Ltd.), which was developed for the evaluation of protein conformational changes in biological tissue [29].

### Transmission electron microscopy (TEM)

Specimens for TEM observation were prepared as follows. First, 2 μL of each insulin material was deposited onto copper grids (200 mesh; Nisshin EM Co., Ltd, Tokyo, Japan) covered with collodion film hydrophilized by an electric glow discharge. After 30 s of deposition, any excess material was blotted out using a filter paper, followed by two deposition–blotting cycles with 20 μL of water and two additional cycles of phosphotungstic acid (25 μL of 1 % w/v). Prior to sample preparation, the staining solution was filtered using a 0.22-μm membrane to remove large crystals. The TEM observation was performed using a Hitachi H-7650 (Tokyo, Japan) at an accelerating voltage of 120 kV.

### Congo Red (CR) assay

The absorbance peak of CR is known to shift from 490 to 510 nm in the presence of the fibrils [30]. Aliquots of the insulin solution (30 μL) were added to an equivalent volume of the CR solution (0.2 mM in PBS) and incubated for 10 min at room temperature. The resulting absorbance values were obtained from a 400–600-nm scan using a multi-label counter (PerkinElmer, Tokyo, Japan).

### Size-exclusion chromatography (SEC)

To detect the insulin monomer, SEC was performed. The gel for SEC (Toyopearl HW-40C from Tosoh Co., Tokyo, Japan) was filled in the column (bed volume 2 mL) and equilibrated with 20 % acetic acid. The molecular weight exclusion limit of the gel was 2.18 × 10^3^ Da according to the certificate of analysis. Protein samples (100 μL) were centrifuged as described above, and the resulting supernatants were loaded onto the column. Elution was performed using the acidic solution, and the protein concentration of each fraction (100 μL) was measured using an ND-1000 Spectrophotometer (NanoDrop Technologies, Inc., Wilmington, DE, USA). Bovine serum albumin (60 kDa) was used as a calibration marker of molecular weight and eluted in fraction nos. 7–8.

## Results

A schematic overview of FEL generation is given in Fig. 1a. Prior to the oscillation of the FEL, the laser was focused right above the sample using a He/Ne beam. The diameter of the laser beam was ~0.5 cm. The materials irradiated by the FEL were analyzed by various methods, as shown in Fig. 1b. The samples were dried for FT-IR analyses and redissolved in water for the CR assay, TEM, and SEC. Irradiation by the FEL tuned to the amide I band (1,620 cm^−1^) was monitored using the power meter (Fig. 1c). The measurements were performed three times, and the power values were found to range from 6.0 to 8.0 mJ/macro-pulse, which resulted in 30.6–40.8 mJ/cm^2^ on the sample each time. The standard deviation of the power value was about one tenth of the average at each measurement. However, for long-term irradiation (more than 1 h), the power tended to decrease. It can be considered that the decrease is due to a reduction in the acceleration voltage, which can be caused by an increase in the temperature of the apparatus itself during the operation.

### Effect of mid-IR FEL irradiation on the disaggregation of insulin fibrils

**Fig. 2:**
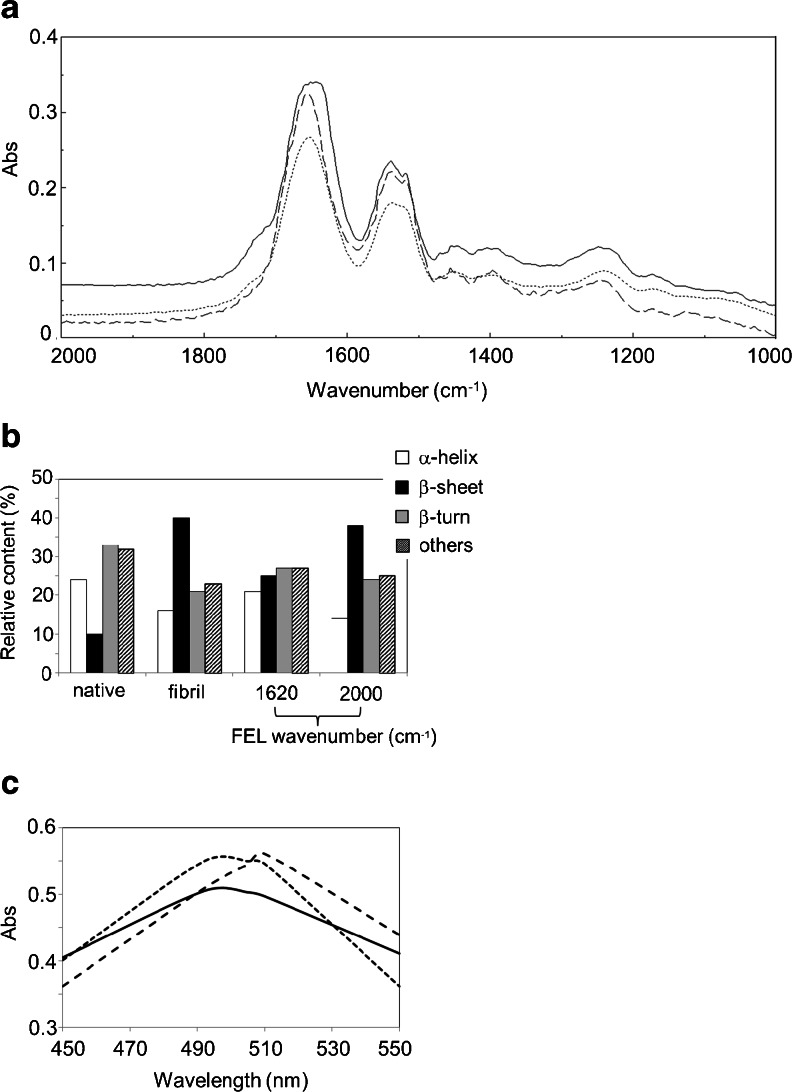
Structural changes in insulin. **a** FT-IR spectra before and after FEL irradiation at 1,620 cm^−1^ for 1 h. The *solid line* represents the spectrum of the insulin fibrils before irradiation, the *dotted line* represents that after irradiation, and the *dashed line* represents the spectrum of native insulin. **b** Secondary structure analyses. Relative contents were calculated based on the protein analysis software (IR-SSE). *Others* indicates the disordered region. **c** CR assay. The *solid line* represents the spectrum of CR (0.2 mM) with native insulin (ca. 2.0 mg/mL), the *dashed line* represents that with insulin fibrils, and the *dotted line* represents that with the fibrils following FEL irradiation

Insulin fibrils were prepared in an acidic solution containing a high concentration of salt as in a previous study [14]. In Fig. 2a, the FT-IR spectrum of the fibrils displayed a broad peak (1,630–1,660 cm^−1^) at the amide I band (solid line), although the main peak appeared at 1,656 cm^−1^ in the native state (dashed line). The β-sheet content in the fibrils was estimated to be around 40 %, whereas it was about 10 % in the native state, by secondary structure analysis (Fig. 2b) that had been developed for the observation of molecular changes of necrotic tissues of murine carcinoma [29]. In contrast, α-helix content in the fibrils was 16 % while that in the native state was 24 %. These secondary structural changes are determined to be caused by the formation of intermolecular β-sheet structures and are consistent with previous data on amyloid fibrils (Tables 1 and 2). Next, the fibrils were placed on the glass slide and irradiated with the FEL which was tuned to 1,620 cm^−1^. After 1 h of irradiation, the broad peak of the fibrils resolved into almost a single peak at 1,652 cm^−1^ (dotted line), and the β-sheet content was reduced to 25 % and α-helix content increased to 21 %. In contrast, irradiation tuned to 2,000 cm^−1^ retained the β-sheet-rich structure of fibrils (β-sheet, 38 %; α-helix, 14 %). These results indicate that a conformational change can occur in insulin fibrils during FEL irradiation at the amide I band, and in particular, the effect of the FEL on the secondary structure is dependent on the output frequency.

The CR assay was also performed to clarify the effect of the FEL on the dissociation of insulin fibrils into the native form (Fig. 2c). The dye is known to bind to amyloid fibrils, and the absorbance peak shifts from 490–500 to 500–510 nm upon binding [30]. While one peak was observed at 492 nm in the case of native insulin bound to the dye (solid line), the peak was shifted to 510 nm when the fibrils were bound to the dye (dashed line). When the dye was mixed with the fibrils after FEL irradiation at 1,620 cm^−1^, the absorbance peak was shifted to near 492 nm, although a slight peak remained at 510 nm (dotted line). This result indicates that non-fibrils were more abundant than the fibrils after FEL irradiation.

### Disaggregated structure of insulin fibrils

**Fig. 3:**
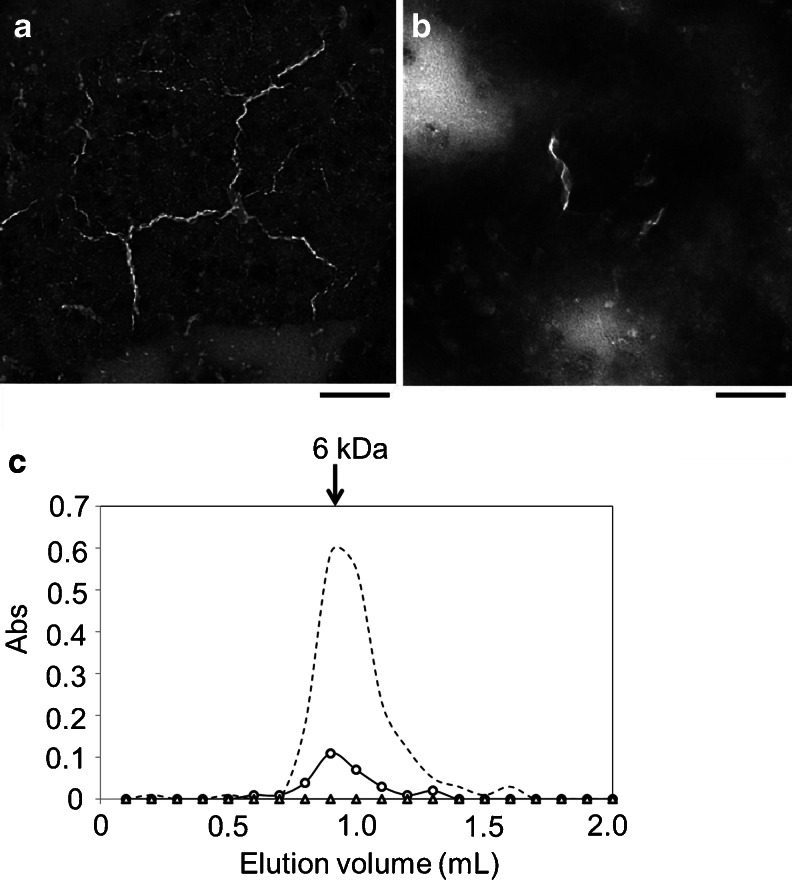
Disaggregated form of insulin fibrils. **a** TEM image of insulin fibrils. *Bar*, 100 nm. **b** Image of disaggregated insulin fibrils. The insulin fibrils were disaggregated in water containing 20 % acetic acid, dried, and redissolved in water for negative staining. **c** Size-exclusion chromatography analysis. The insulin sample was fibrillated, and the supernatant after centrifugation was loaded on the gel and eluted (*triangle*). The fibrils were disaggregated and centrifuged, and the supernatant was loaded (*circle*). The *dotted line* indicates the standard insulin monomer (6 kDa)

To elucidate the morphology and structure of the disaggregated form of the insulin fibrils, we analyzed the disaggregated insulin fibrils using TEM and SEC (Fig. 3). Insulin fibrils were prepared and disaggregated on the glass slide as described above (original protein concentration, 2.0 mg/mL in 20 % acetic acid). As shown in Fig. 3a, several thin strings were observed. Each string appears as a helical structure rather than a straight line. The lengths of the fibrils were 100–300 nm and their widths were about 10 nm. These thin strings decreased substantially after disaggregation (Fig. 3b). While short-length helical strings remained, long helical fibrils disappeared. These TEM observations support our observation that the fibril structure is converted into the non-aggregated form by the FEL. The disaggregated material was next analyzed by SEC (Fig. 3c). In this chromatography system, a standard sample of insulin monomer was eluted in fraction no. 9 (dotted line). When the supernatant after fibrillation was loaded on the column, no peaks were detected (triangle). On the other hand, when the supernatant of the disaggregated fibrils was loaded, a monomer peak was detected at fraction no. 9 (circle). The extent of recovery was calculated to be about 20 % of the total protein based on the absorbance (1.0 Abs = 1.0 mg/mL). Remarkably, no oligomer forms were detected in the column (large proteins such as BSA with molecular weights greater than 10 kDa must be eluted before the insulin peptide). This result indicates that FEL irradiation can dissociate the insulin fibrils into the monomeric form, without producing any high molecular weight oligomers.

## Discussion

Mid-infrared free-electron lasers in the biological and medical fields have been used mainly for tissue ablation in surgery [1–12]. Although the effect of the FEL on protein structure has been accepted in the course of those studies, detailed conformational changes of protein folding at the sub-nanometer level have not yet been studied. We have demonstrated that the FEL tuned to the amide band can dissect protein aggregates into the monomeric form, and this result indicates that FEL irradiation of the protein affects the protein folding machinery. A common feature of amyloid fibrils is that they are very stable under physiological conditions. In the case of Aβ, the fibrils can be accumulated in the brain tissue of patients with Alzheimer’s disease [31]. Although treatment of the amyloid fibrils does not necessarily lead to the direct therapy of the diseases, exploring the structural changes of fibrils into the globular forms is very important to understand the protein folding mechanism. Booth et al. first showed that the amyloid fibrils of lysozyme could be refolded under denaturation conditions [32]. This is in some ways a landmark result because the robust fibrils are flexible and dynamic in solution. In contrast, we found that lysozyme fibrils could be refolded into the native state in salt-free neutral water and that mid-IR FEL irradiation tuned to amide bands could promote the refolding of the fibrils at mild temperatures (37 °C) [14]. Using a similar method, we found that insulin fibrils could be refolded into the monomeric form in this study. The refolding mechanism under FEL irradiation is probably different from that resulting from the use of denaturants. It can be estimated that FEL irradiation at the amide band heats the fibrils and the surrounding water, driving the dissociation of the fibrils and refolding them into the native state. We believe that non-covalent bonds between β-sheet structures can be affected by FEL irradiation. Vaporization of water is also suggested to be a driving force for fibril dissociation. However, the refolding efficiency of insulin was lower than that of lysozyme. That is, the β-sheet content of the lysozyme fibrils recovered almost completely to a level similar to that of the native state after FEL irradiation at the amide I band [14], whereas that of insulin fibrils did not fully recover (Fig. 2b). Such a tough structure for the insulin fibrils was also evident from SEC analysis (Fig. 3c). These results confirm that insulin fibrils have a more robust structure in solution than do lysozyme fibrils.

Amyloid fibrils are formed by diverse polypeptides and are deposited in many tissues of various organs during amyloidosis. However, the mechanism by which amyloid fibrils form and the strategy to treat amyloidosis in diseases such as myeloma remain to be established [33]. It can be expected that the above FEL irradiation system should be applied for treatment of those diseases, although the present system requires a mid-scale photon factory. Instead, a more compact irradiation technology such as an endoscope combining an optical fiber, in which oscillation wavelength should be tuned to the mid-infrared amide region, may be necessary for the clinical application. This technological method is now under study.

In conclusion, the above results confirm that the FEL irradiation yields the monomer from amyloid-like protein fibrils. We believe that not only amyloid fibrils but also other protein aggregates in biological system can be altered by FEL irradiation. Protein fibers have high-order structures containing hydrophobic intermolecular clusters and a hydrogen bond network similar to amyloid fibrils. In the future, FEL will be also applied to the disaggregation of various protein fibrils related to several biological phenomena.

## Abbreviations

CR: Congo Red
FT-IR: Fourier transform infrared spectroscopy
Mid-IR FEL: Mid-infrared free-electron laser
PBS: Phosphate-buffered saline
SEC: Size-exclusion chromatography
TEM: Transmission electron microscopy
